# Effective and Safe Use of Glucocorticosteroids for Rescue of Late ARDS

**DOI:** 10.1155/2017/6740532

**Published:** 2017-02-26

**Authors:** Paolo Diana, Dustin T. Money, Michael G. Gelvin, Nadia Lunardi

**Affiliations:** ^1^Anesthesiology, Università degli Studi di Padova, Padua, Italy; ^2^Cardiovascular Perfusion, University of Virginia Health System, Charlottesville, VA, USA; ^3^Anesthesiology, University of Virginia Health System, Charlottesville, VA, USA

## Abstract

We describe a case of severe refractory hypoxemia requiring prolonged extra corporeal membrane oxygenation (ECMO) support in a case of postpartum acute respiratory distress syndrome (ARDS). The clinical course was marked by persistently poor lung compliance and several complications of ECMO, that is, significant hemolysis, hemothorax, and intracranial bleeding. We report marked improvement of lung mechanics and respiratory function, leading to accelerated separation from ECMO, following rescue administration of low dose methylprednisolone 24 days after the onset of ARDS. Corticosteroid treatment was safe and well tolerated. In contrast with the conclusions of the 2006 ARDS Network trial, our report establishes a case in support of the use of low dose methylprednisolone as a safe and effective rescue treatment option in selected subsets of patients with nonresolving ARDS.

## 1. Introduction

Debate regarding the efficacy and safety of glucocorticosteroids (GC) in the treatment of septic shock and ARDS has continued over many years. While the use of low dose GC was initially incorporated into the 2004 Surviving Sepsis campaign guidelines, the latest guidelines (2012) suggest not to use GC to treat adult septic shock patients, unless adequate fluid resuscitation and vasopressor therapy are unable to restore hemodynamic stability (grade 2C evidence) [1]. Likewise, while the 2006 ARDS Network trial found no difference in mortality at 28 and 180 days following treatment with low dose GC and thus discouraged their routine use in ARDS [2], the most recent consensus statement from the American College of Critical Care Medicine (2008) suggests that moderate dose GC should be considered in patients with severe ARDS (grade 2B evidence) [3].

## 2. Case Presentation

C. A. was a 25-year-old pregnant female, with a history of cigarette smoking, gestational diabetes, and preeclampsia, who underwent an uneventful cesarean section at an outside hospital secondary to failure to progress. However, on postpartum day 3 she developed rapidly progressive refractory hypoxemia. Despite maximal mechanical ventilation support, deep sedation with paralysis and prone positioning, her oxygenation continued to worsen. In the setting of PaO_2_ : FiO_2_ ratio below 100, she was transferred to the University of Virginia Medical Center for initiation of ECMO on postpartum day 10. Upon arrival, her arterial blood gas was: PH 7.35/PCO_2_ 45.8 mmHg/PaO_2_ 47.9 mmHg on pressure control ventilation at 24 cmH_2_O, PEEP 20 cmH_2_O, FiO_2_ 100%, with tidal volumes between 130 and 200 ml and a plateau pressure of 38 cmH_2_O. A 27 French Avalon Elite cannula was placed in the right internal jugular vein and ECMO support was quickly instituted. Despite multiple attempts at repositioning the cannula, as well as judicious volume resuscitation, ECMO flows above 3.5 L/min could not be obtained. This resulted in PaO_2_ and hemoglobin oxygen saturation levels as low as 40 mmHg and 77%, respectively, in the following 24 hours. Protective lung ventilation was maintained according to the ARDSNet protocol. Inhaled nitric oxide use was attempted but discontinued shortly after due to carboxyhemoglobinemia and lactic acidosis. On ECMO day 2, C. A. developed a hemothorax that required surgical hemostasis via a thoracotomy. A head CT scan on ECMO day 6 showed a 5 mm-wide hemorrhage in the genu of the corpus callosum. On ECMO day 10, C. A. was deemed to not be a candidate for lung transplantation.

**Figure 1:**
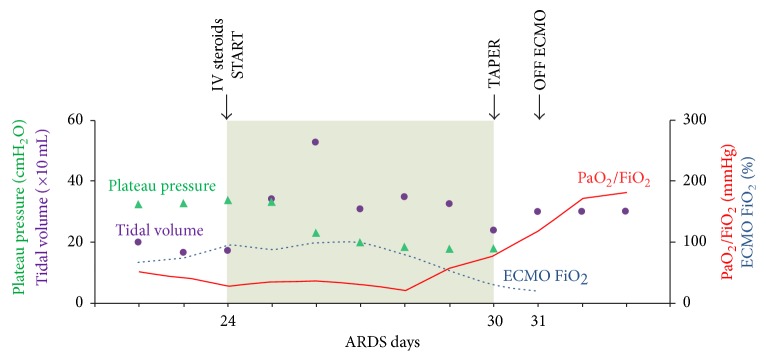


After 16 days on ECMO and 24 days from onset of ARDS symptoms, decision was made to attempt a rescue therapy with low dose GC. A loading dose of methylprednisolone (MP, 1 mg/kg) was started, followed by a continuous infusion (1 mg/kg/day), based on the protocol by Meduri and colleagues [4]. As shown in Figure 1, a marked improvement in lung compliance was noted within 48 hours, as reflected by a significant increase in tidal volume (initially between 130 and 200 ml on Bi-Vent at *P*_high_ 32 cmH_2_O and *P*_low_ 0 cmH_2_O, increased above 300 ml after MP on the same ventilation settings). This was closely followed by a significant decrease in plateau pressures (from 32–34 cmH_2_O to below 20 cmH_2_O). PaO_2_ : FiO_2_ ratio lagged behind and was noted to improve after 4 days of MP infusion. After 7 days of low dose MP our patient's overall respiratory function had dramatically improved and ECMO could be discontinued. C. A. was discharged to a long term care facility to complete weaning from mechanical ventilation on hospital day 57, after 23 days on ECMO. She is currently living at home without need for oxygen supplementation.

## 3. Discussion

We report herein accelerated improvement of lung mechanics and overall respiratory function, allowing for rapid separation from prolonged ECMO, after late administration of low dose methylprednisolone in a postpartum patient with persistent ARDS.

Although translational research has established a strong association between dysregulated systemic inflammation and delayed resolution of ARDS, the results from clinical studies are contradictory. For example, the ARDS Network trial [2] found no difference in mortality at 28 and 180 days in patients with ARDS treated with moderate dose MP. Importantly, the authors also reported increased mortality in patients who received MP more than 14 days after the onset of ARDS, concluding that late steroid therapy may increase the risk of death in persistent ARDS. Their conclusions received considerable criticism in the aftermath, because of the small number of patients with late ARDS included in the study, the uncharacteristically low mortality of control patients (that is, 8% in control versus 36% in ARDS patients), the significant differences in patient baseline characteristics, and the loss of mortality difference after adjusting for imbalances at baseline [5, 6]. On the other hand, a recent analysis of individual patient data from four randomized controlled trials concluded that GC treatment accelerates resolution of ARDS, shortens time to achievement of unassisted breathing, and increases mechanical ventilation- and ICU-free days, without increasing the risk of infection [7]. Based on these updated findings, several experts in the field have called for a shift in the focus of clinical trials from mortality as the golden standard of outcome parameters to broader and more meaningful clinical measures that may better describe quality of survival, that is, PaO_2_ : FiO_2_ ratio, number of mechanical ventilation- and ICU-free days, and other healthcare utilization indexes [8, 9].

In the case of our patient, MP treatment was carefully undertaken understanding fully well the implications of the ARDS Network trial, after failure of conventional treatments, in the face of continued challenges with oxygenation and systemic anticoagulation, and after the possibility of a lung transplant had been excluded. During MP administration, careful infection surveillance was implemented with daily differential white blood counts, biweekly percutaneous blood and urine cultures and weekly bronco-alveolar lavages. Gastrointestinal protection was maintained by continuous infusion of high dose proton pump inhibitors, and amylase/lipase levels were monitored weekly to rule out steroid-induced pancreatitis. C. A. did not develop nosocomial pneumonia or any positive cultures during MP treatment. Blood glucose levels were effectively treated by a computer-directed method of inpatient glucose management. Paralytics were discontinued shortly after MP initiation, given the remarkable improvement in lung compliance and oxygenation.

A limitation of our report is that histological parameters of lung inflammation-fibrosis and levels of plasma and bronchoalveolar lavage (BAL) markers of inflammation-fibroproliferation could not be obtained before and after MP treatment. In fact, IL-6, TNF*α*, and type III procollagen are known to be elevated in the BAL and plasma of patients with ARDS, and their levels are known to undergo rapid, progressive, and significant decrease in those who experience clinical improvement following GC administration [10]. In the absence of these measurements, it cannot be completely ruled out that factors other than MP administration may have been responsible for C. A.'s accelerated ARDS resolution. Nonetheless, the observation of a clear temporal correlation between initiation of MP and a sizable improvement of respiratory function—in the absence of any other changes in treatment—argues in favor of the notion that MP-induced suppression of dysregulated lung inflammation is what led to a breakthrough in our patient's lung disease.

Taken together, our observations support the argument that low dose methylprednisolone may be an effective and safe treatment option in subsets of patients with persistent ARDS, with concomitant implementation of intensive measures for prevention and early detection of infections and other potential steroid-related side effects.

## References

[1] Dellinger R. P., Levy M. M., Rhodes A. (2013). Surviving sepsis campaign: international guidelines for management of severe sepsis and septic shock: 2012. *Critical Care Medicine*.

[2] Steinberg K. P., Hudson L. D., Goodman R. B. (2006). Efficacy and safety of corticosteroids for persistent acute respiratory distress syndrome. *New England Journal of Medicine*.

[3] Marik P. E., Pastores S. M., Annane D. (2008). Recommendations for the diagnosis and management of corticosteroid insufficiency in critically ill adult patients: consensus statements from an international task force by the American College of Critical Care Medicine. *Critical Care Medicine*.

[4] Meduri G. U., Golden E., Freire A. X. (2007). Methylprednisolone infusion in early severe ARDS: results of a randomized controlled trial. *Chest*.

[5] Meduri G. U., Marik P. E., Chrousos G. P. (2008). Steroid treatment in ARDS: a critical appraisal of the ARDS network trial and the recent literature. *Intensive Care Medicine*.

[6] Meduri G. U., Marik P. E., Pastores S. M., Annane D. (2007). Corticosteroids in ARDS: a counterpoint. *Chest*.

[7] Meduri G. U., Bridges L., Shih M.-C., Marik P. E., Siemieniuk R. A. C., Kocak M. (2016). Prolonged glucocorticoid treatment is associated with improved ARDS outcomes: analysis of individual patients’ data from four randomized trials and trial-level meta-analysis of the updated literature. *Intensive Care Medicine*.

[8] Bein T., Briegel J., Annane D. (2016). Steroids are part of rescue therapy in ARDS patients with refractory hypoxemia: yes. *Intensive Care Medicine*.

[9] Meduri G. U. (2015). Diffuse alveolar damage in nonresolving ARDS provides support for prolonged glucocorticoid treatment. *Intensive Care Medicine*.

[10] Meduri G. U., Headley S., Tolley E., Shelby M., Stentz F., Postlethwaite A. (1995). Plasma and BAL cytokine response to corticosteroid rescue treatment in late ARDS. *Chest*.

